# Turning ideas into action: A framework for cardiothoracic trainees and surgeons to launch translational clinical trials

**DOI:** 10.1016/j.xjon.2025.09.005

**Published:** 2025-09-10

**Authors:** Zyriah Robinson, Jessica B. Briscoe, AlleaBelle Bradshaw, Lisa Fornaresio, Jennifer S. Lawton

**Affiliations:** a Clinical Research Unit, Division of Cardiac Surgery, Johns Hopkins University School of Medicine, Baltimore, Md; b Department of Surgery, Division of Cardiac Surgery, Johns Hopkins University School of Medicine, Baltimore, Md

**Figure:**
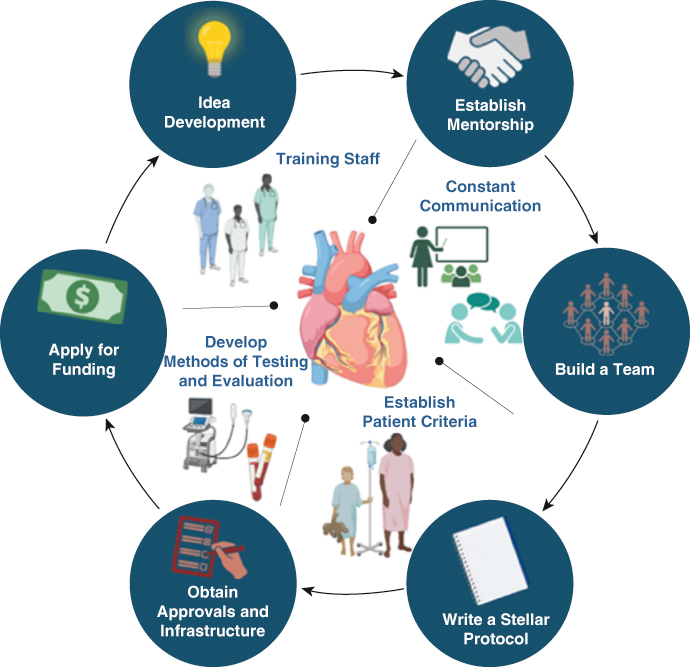
Framework for clinical trial initiation. Created in BioRender (https://BioRender.com).

**Central Message**

Idea development is the foundation of a clinical trial, in which mentorship, preparation, and education empower budding cardiac surgeon-scientists to transform challenges into opportunities for advancing patient care.

**Perspective**

Initiating investigator-led clinical trials demands significant effort, clear communication, and regulatory knowledge, and education in these areas is often lacking in surgical training. We present a framework to guide young surgeon-scientists in developing high-quality, effective clinical trials.

Initiating a clinical trial is highly rewarding and can lead to substantial benefits for cardiothoracic surgery patients. Learning to navigate this process poses complex challenges for which the overwhelming majority of cardiothoracic surgeons have no training or preparation. While the first author's perspective is shaped by a nonclinical background (with the aspiration to become a surgeon), the operational and regulatory hurdles described are common to all early-stage trialists. For a young surgeon, these challenges may be even more pronounced, as clinical training rarely equips one with the skills needed to lead a clinical trial. This guide aims to offer practical insights that are broadly applicable, regardless of one specific training path.

With no background in research, my path into clinical trials began with curiosity and an opportunity. I earned my Bachelor of Arts in Biology with a minor in Sociology. After graduation, I became interested in clinical research but was unsure how to get started. When an opportunity arose in cardiac surgery research at Johns Hopkins, I saw a chance to not only gain new skills but also to contribute to something meaningful. This experience opened my eyes to the complexities of clinical trials. My future goals involve building a career at the intersection of medicine and research.

Starting investigator-initiated studies requires substantial effort, dedication, clear communication, and a deep understanding of regulatory processes. Clinical trials led by surgeons are rare, largely due to the lack of formal education relevant to initiating trials.^1^ Few resources are available to provide guidance to the novice surgeon-trialist, including seminars and courses (Table E1); as a result, many surgical clinical trials suffer from inefficiencies, and research waste is a well-documented issue in the field.^2^

Randomized clinical trials (RCTs) in surgery are difficult to design and implement, often resulting in small trial sizes designed to detect large effects, which many fail to do.^3^ Without proper guidance, even the most motivated surgeon-scientists may struggle to launch successful studies independently. In this Young Surgeon's Note, we discuss a framework for young surgeon-scientists to prepare for high-quality, effective clinical trial development (Table 1). We provide insight regarding regulatory navigation, team assembly, and trial infrastructure that can be adapted to device and multicenter studies.

**Table 1:** Regulatory checklist for initiating a clinical trial

Regulatory item	Considerations
Protocol development	Finalize study protocol, including objectives, design, and procedures
Informed consent form	Ensure language is participant-friendly and includes all required elements
FDA IND/IDE application	Forms to complete: 1571, 1572, 3674, etc
IRB	Vulnerable populations, risk-benefit ratio, informed consent process
Trial registration: ClinicalTrials.gov	Registered before enrollment; maintenance of ClinicalTrials.gov record throughout the trial, trial results dissemination
DSMB	Creation of DSMB charter, ongoing monitoring of enrolled patients and their data, adverse event adjudication and reporting
Delegation of authority and training logs	Assign study responsibilities and document staff training
IDS/IPM	Drug or device accountability

*FDA*, Food and Drug Administration; *IND*, Investigational New Drug; *IDE*, Investigational Device Exemption; *IRB*, Institutional Review Board; *DSMB*, Data Safety Monitoring Board; *IDS*, Investigational Drug Services; *IPM*, Investigational Product Management.

## Developing an Idea

**Figure 1:**
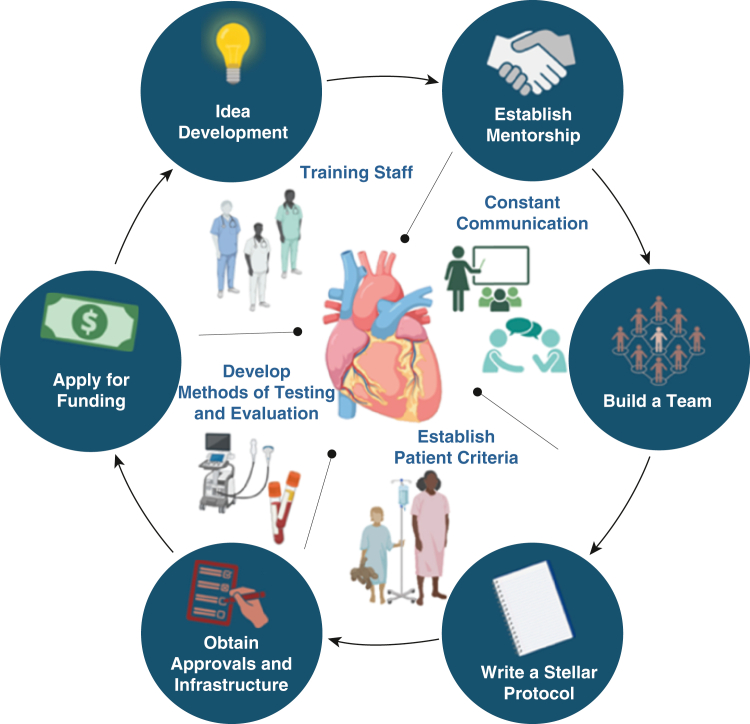
Framework for clinical trial initiation. Created in BioRender (https://BioRender.com).

Developing an idea for a clinical trial involves identifying a specific medical issue or unanswered question that could significantly impact patient care (Figure 1). For me, this process became clear when I started working in Cardiac Surgery Research. I quickly realized that while there were many important clinical questions, translating them in a well-structured trial was far more complex than I had initially expected. The process begins with a thorough review of existing research to ensure the idea is relevant and addresses a genuine gap in knowledge or treatment options. Researchers must define the trial's objectives, whether to evaluate the safety and efficacy or compare the effectiveness of a treatment or intervention. The next step is designing the trial, selecting the appropriate patient population, determining endpoints, and outlining a study protocol. Starting a clinical trial requires more than just a compelling idea—it must be grounded in solid scientific rationale. The research waste from surgical trials is largely due to the misinterpretation or use of inadequate scientific evidence.^2^ Ensuring that an idea is both feasible and impactful is one of the biggest challenges but also one of the most rewarding aspects of the research process.

## Background of a Solid Science Foundation

The hypothesis should be based on sound basic science and robust preclinical evidence. Identifying research that is truly ready for clinical translation can be a daunting task. In my time working in cardiac surgery research, I have witnessed firsthand the challenges in assessing preclinical findings and determining how they can be effectively translated into real-world treatments and solutions. Many basic science studies involving in vitro and preclinical animal research fail to be successfully translated into human trials and clinical practice.^4^^,^^5^ Understanding these challenges firsthand has reinforced the importance of building a strong scientific foundation before moving forward with a clinical trial.

## Developing a Research Question and Writing the Study Protocol

A clinical trial should be focused on answering a specific question. One framework to aid researchers in developing a focused clinical trial is PICO (population or patient, intervention, comparison, and outcome). For population or patient, the group of patients should be specific with demographic information and medical history identified. For intervention, the therapy, treatment, drug, or surgical method should be clearly defined. For comparison, the group used for comparison should be determined. Finally, the outcome should be measurable, distinct, and translatable to the larger population (eg, operative mortality, long-term disability, etc).^6^

Developing a clinical trial takes careful planning and attention to detail, but in my experience, it is rarely a straightforward process. I quickly realized that even the best-designed studies face unexpected hurdles. Dr Lawton's lab had extensive preclinical supporting data, and we wanted to begin translation to humans. We were encouraged and felt that it was time because National Institutes of Health (NIH) reviewers of our grants and editorials about our papers suggested moving forward with a clinical trial. The trial that we proposed would evaluate adenosine triphosphate–sensitive potassium channel opener diazoxide in cardioplegia to reduce myocardial stunning after cardiac surgery (NCT06308107; Safety and Feasibility of Hyperkalemic Cardioplegia with Diazoxide in Cardiac Surgery [CPG-DZX] trial).

One of the greatest challenges that we faced was meeting the US Federal Drug Administration (FDA) requirements for an Investigational New Drug (IND) application. An IND application is a request submitted to the FDA to start a clinical trial of a new drug in humans. Even though this drug was previously approved for 2 other uses (orally to treat symptomatic hypoglycemia and intravenously for hypertension), a new FDA IND application was required for the intracoronary administration. The FDA mandated teratogenicity testing, which would have significantly delayed our trial. To work around this, we adjusted the protocol to include strict birth control requirements for participants. It was a small but critical change that allowed us to move forward without costly and time-consuming additional testing.

Securing necessary documentation, including the Certificate of Manufacturing and impurities testing from Pfizer (the current manufacturer of the drug), also proved more complex than expected. Each step required multiple rounds of communication, approvals, and adjusted timelines. We made major changes to meet FDA regulations on serious adverse events (SAEs), which meant revising the protocol several times. Because the drug currently is in clinical use only in New Zealand and Australia, international shipping restrictions added another layer of complexity and cost.

Our experience for this particular trial relates to the process for obtaining an IND. Surgical trial also may require an Investigational Device Exemption (IDE). An IDE application is a request submitted to the FDA to start a clinical trial of a new device intended for use in humans. An IDE application has many similarities to an IND application in terms of the type of information required (safety, manufacturing, etc).

Preparing the study protocol requires the determination of the target demographic and disease or condition to be studied, ensuring alignment with the research objectives. Establishing eligibility criteria is essential to select participants most likely to benefit from and contribute to the study's success while minimizing risks. The protocol must outline the testing and evaluations necessary to collect data, including laboratory assessments. The protocol should include details of the day-to-day requirements of recordkeeping, drug preparation, drug storage, and data maintenance.

Determining the sample size required for enrollment should be done in collaboration with a statistician. Many academic institutions have an office of clinical research or statisticians available for consultation for these estimations as well as overall study design (randomized, crossover, adaptive), primary and secondary endpoint determinations, outcome timepoint assessments, power considerations for primary and secondary outcomes, gender and diversity considerations of participants, and handling of missing data.

Building a multidisciplinary team is essential to the successful execution of a clinical trial. Engaging experts in pharmacy, biostatistics, clinical operations, and regulatory affairs early in the process ensures that critical elements (including appropriate blinding procedures, placebo or active comparator packaging, and a clearly defined plan for unblinding) are addressed thoughtfully. These components can have significant implications for trial design, logistical feasibility, and regulatory compliance, and they benefit from the insights of team members with specialized expertise.

## Developing a Budget and Securing Funding

Funding is essential to cover the costs associated with research, staffing, and infrastructure (Table E2). The NIH offers various biomedical and public health research grants, including R61/33 Grants, which support early-phase clinical trials with pathways to additional funding for subsequent phases. Industry partnerships can provide significant financial support if the pharmaceutical company is interested in advancing a specific intervention (drug or device). The Cardiothoracic Surgery Trials Network (CTSN) is a specialized NIH network that funds collaborative research in cardiothoracic surgery. Additional potential funding sources include philanthropic awards or awards from foundations that support cardiothoracic research.

## Identifying and Developing a Study Team

Building an effective study team is a critical step to ensuring a successful clinical trial. Building a team begins with identifying key roles and responsibilities required for the trial, including the principal investigator (PI), co-investigators (Co-I), study coordinators, data managers, and regulatory specialists. The PI and CO-I are responsible for identifying good candidates and conducting clinical conversations with participants. Coordinators help manage day-to-day study activities and participant interactions. The Data Manager is responsible for cleaning and monitoring the data to ensure accuracy and integrity. The Regulatory Specialist oversees regulatory compliance, including Institutional Review Board (IRB) submissions and FDA documentation. Each member should have the appropriate training and certificates to ensure compliance. Clear communication and collaboration are essential, and fostering a team environment in which everyone feels valued and aligned with the study's goals can significantly impact the trial's efficiency and success.

## Obtaining Approval from the FDA and the Institutional Ethics Committee

A clinical trial can involve testing a drug, a device, a technique, or a process. The regulatory oversight and approval process may vary depending on the type of investigations. Here we focus on the evaluation of a drug, because that is the experience that inspired this manuscript (translation of preclinical work to a Phase I safety and feasibility trial in humans). An IND application to the FDA is essential to begin human testing in the United States and must include detailed information about the drug's composition, manufacturing, and intended clinical protocols. The FDA ensures the safety and rights of trial participants and assesses the scientific rationale of the proposed study. Key components of an IND application include preclinical data, manufacturing information, clinical trial protocol, and relevant forms, such as the FDA Form 1571 and Form 3674. The FDA has 30 days to review an IND application. Investigators should plan to expect several revisions and requests for additional information before approval is given. In our experience, the review process took longer than expected (2 years). The FDA staff managing the IND turned over several times during the process, leading to additional delays. Additional delays were related to determining the original manufacturer of the drug, identifying the chain of information to the current manufacturer, obtaining confidential disclosure agreements to obtain CMC (chemistry, manufacturing, and controls) and CoA (certificate of analysis) information regarding the drug, and performing impurity testing on the drug to meet the FDA requirements. In our case, the thin-layer chromatography analysis performed by the manufacturer was not acceptable, and thus we paid for further analysis via high-performance liquid chromatography.

Submitting an IRB application is another required step before starting trial enrollment. Considerations for a thorough IRB review include the potential involvement of vulnerable populations, non–English-speaking participants, consent for future use of biological specimens, and procedures for enrolling individuals unable to provide consent for themselves. An IRB is typically a group that acts as an ethics committee to ensure that research involving humans is conducted according to federal regulations, state laws, and institutional policies. This application will come back with multiple revisions to ensure that investigators answer and address all questions or concerns from the IRB. The total process at Johns Hopkins took 6 months before approval was finally granted.

All investigator-initiated trials must comply with FDA regulations under 21 CFR Part 312, which outlines the requirements for IND or IDE applications, safety reporting, recordkeeping, and sponsor-investigator responsibilities. Early collaboration with regulatory experts or institutional IND/IDE offices can help ensure that these obligations are met.

Adherence to Good Clinical Practice (GCP) guidelines is essential for ensuring participant safety, data integrity, and regulatory compliance. GCP principles provide a standardized framework for the ethical and scientific conduct of clinical research and are expected in both industry-sponsored and investigator-initiated trials. Training in GCP is typically required by institutions and should be completed prior to trial initiation.

A critical component of a clinical trial is a Data Safety Monitoring Board (DSMB), which should be verified as being thorough and without any conflicts of interest. The DSMB charter defines the board's structure, responsibilities, and the guidelines that its members will follow throughout the trial. At our institution, the principal investigator is responsible for assembling the board. The DSMB monitors the enrolled patients and their data, ensures safe and ethical practices are conducted, and determines the need to report adverse events or protocol deviations to the FDA and IRB.

Curating a relationship with Investigational Drug Services (IDS) was essential for the smooth conduct of our trial. IDS is an internal institutional department that is used to manage the drug supply and distribution throughout the trial. Navigating this relationship can be complicated, especially as it requires institutional procedures and regulations. At times, there were struggles with communication and coordination that slowed overall progress and presented acute challenges when enrolled patients underwent surgery.

In addition to drug logistics, coordinating required testing such as labs and echocardiograms, presented additional challenges. Scheduling and completing these procedures within protocol-specified windows required constant vigilance and real-time communication. Building a strong working relationship with IDS and maintaining open communication among all teams is crucial to overcoming obstacles and ensuring smooth trial execution.

## Registering the Trial

Registering a clinical trial involves submitting detailed information about the trial to a recognized registry, such as ClinicalTrials.gov. The registration provides a snapshot of the study's objectives, design, population, interventions, eligibility criteria, primary and secondary endpoints, and an anticipated timeline. At Johns Hopkins, the IRB requires registration of the study on ClinicalTrials.gov before enrollment. ClinicalTrials.gov is a publicly accessible site at which patients can search for trials and even request to be included, helping increase participation in research.

## Informed Consent

There are often gaps in how informed consent is approached and communicated, particularly in distinguishing the goals of research from those of clinical care. Obtaining consent from a patient for a clinical trial is a critical process to ensure that the patient fully understands the study and their role in it. This begins with a clear and patient-centered conversation in which the researcher explains the trial's purpose, procedures, potential benefits, randomization assignments if applicable, use of their personal data, and risks in a language that is easy to understand. It is essential to give the patient ample time and opportunity to ask questions and address concerns. The patient should feel no pressure to participate and be assured that their care will not be affected if they choose not to enroll. Transparency and trust are important, and researchers must confirm that the patient comprehends the information before signing the consent form.

Approaching a patient who is nervous about enrolling in a study requires empathy, patience, and clear communication. It is essential to listen actively to understand the patient's specific reasons behind any apprehension and concerns. Addressing concerns with factual, transparent information about the study, highlighting the study's purpose and potential benefits, is key. It is also important to allow time for the patient to read the consent form, process the information, and ask any questions. Reassuring the patient that participation is entirely voluntary and that they can withdraw at any time without any impact on the standard of care procedures helps ease worries. Finally, offering to connect the patient with the clinician for further questions can provide additional reassurance.

## Constant Communication and Patient Enrollment

Effective team communication and collaboration will ensure alignment across various hospital systems, departments, and teams. Every team and department involved has its unique workflow and protocol, and understanding these differences is key. Our team frequently worked with multiple departments, including the operating room, intensive care unit, stepdown unit, Advanced Practice Providers, and the echocardiography team. This work involved creating educational reminder sheets, arriving in the operating room early to alert staff of the patient's participation, and attending staff meetings to foster collaboration. Strong communication enables teams to troubleshoot challenges effectively and be flexible to pivot as needed.

## Identifying Mentorship

Mentorship is vital when conducting a clinical trial, particularly for early-career investigators navigating regulatory, logistical, and methodological complexities for the first time. Identifying a mentor involves seeking experienced professionals with expertise not only in the scientific area relevant to the study but also in clinical research operations. A strong mentor provides critical guidance on best practices, helps troubleshoot challenges, and shares insights drawn from prior trial experience—often including missteps that can serve as teachable moments. Mentors can offer valuable feedback on protocol development, study design, data analysis, and team dynamics and can help junior investigators anticipate and manage institutional and regulatory hurdles.

## Conclusions

**Figure 2:**
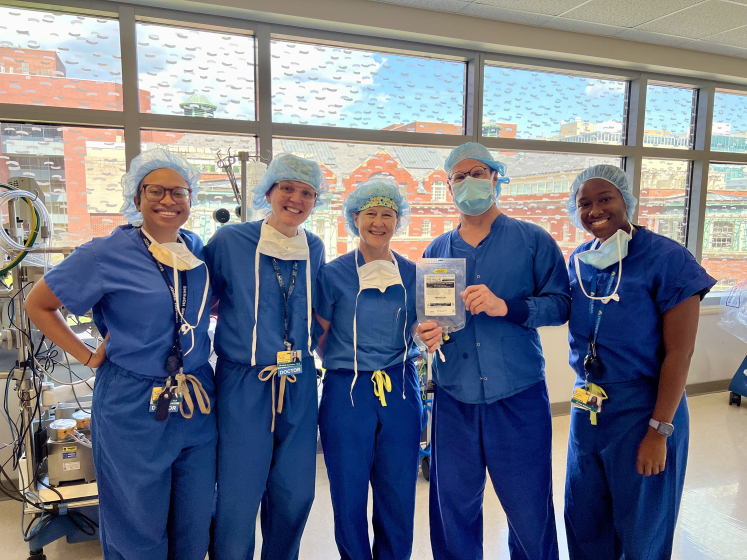
Research team after enrolling subject 1 (from left to right: Jessica Briscoe, AlleaBelle Bradshaw, Jennifer Lawton, Herbert Harness, and Zyriah Robinson).

Initiating a clinical trial takes significant effort, patience, and meticulous planning. We successfully enrolled all 30 patients for our Phase 1 trial, meeting our original recruitment targets, timelines, and budget. This is a major milestone that reflects the persistence, collaboration, and attention to detail that our team maintained throughout the process. This journey is incredibly rewarding (Figure 2); it not only advances science and improves patient care but also provides a wonderful learning opportunity for a future surgeon. By building a strong foundation of scientific rationale, fostering effective collaboration, and adhering to regulatory standards, researchers can successfully navigate the complexities of a clinical trial and contribute to meaningful medical progress.

## Conflict of Interest Statement

The authors reported no conflicts of interest.

The *Journal* policy requires editors and reviewers to disclose conflicts of interest and to decline handling or reviewing manuscripts for which they may have a conflict of interest. The editors and reviewers of this article have no conflicts of interest.

## Figures and Tables

**Table E1:** Current educational guidance for surgeon-scientists on initiating a clinical trial

Reference	Focus	Pitfalls
Roland et al^E1^	Clinical trial course	Open only to University of Texas MD Anderson Cancer Center surgical oncology fellows
Fleshman^E2^	Guidance on initiating clinical research	Focus on colon and rectal surgeon investigators
McDonald et al^E3^	Developing investigator-initiated trials in surgical oncology	Focus on processes that may be specific to surgical oncologists
Scoglio and Fichera^E4^	Broad review of a developing clinical research program	Not detailed; no step-by-step guidance
American College of Surgeons^E5^^,^^E6^	Overview on starting a clinical trial	Coursework must be purchased
American Association for Thoracic Surgery Clinical Trial Methods course	Intensive and interactive training program to acquire critical skills in effective clinical trial design and implementation, with a focus on preparation of clinical trial proposals and funding	A 2-d intensive course in a different city every year; information not posted in advance
Society of Thoracic Surgery Clinical Trials Curriculum	Modules that provide an overview of 4 study design methodologies	Focus is only on study design, not implementation or funding
FDA Continuing Education Programs	Supports interprofessional continuing education for health professionals.	Continuing education credits for activities at no cost to learners through workshops, courses, seminars
Introduction to the Principles and Practice of Clinical Research (IPPCR)	Introduction to the principles and practice of clinical research	Online course offered annually at no cost
CITI Good Clinical Practice (GCP)	Basic and refresher courses that provide essential good clinical practice training for research teams involved in clinical trials	Cost dependent on institutional affiliation

**Table E2:** Awards and funding available to cardiothoracic surgeon researchers

Award/funding	Amount	Eligibility	Website
Thoracic Surgery Foundation Research Award	$85,000/y for up to 2 y (total $170,000)	Early investigators must be certified by the American Board of Thoracic Surgery by the start of the grant and within 7 y∗ of their first faculty appointment.	https://www.thoracicsurgeryfoundation.org/awards/tsf-research-award Early-career phase
Patient-Centered Outcomes Research Institute (PCORI)	Varies, up to $120 million	Does not fund individual investigators. Does fund private and public sector organizations and US organizations.	https://www.pcori.org/fundingopportunities Career phase not applicable
American Heart Association (AHA)	Varies, up to $1 million	Largely based on the principal investigator’s career stage.	All career phases
AHA Career Development Award	$77,000/y, including 10% institutional indirect costs (total $231,000)	Must have attained faculty appointment by the time of award activation; at least 10% effort to the award; can have a K award concurrently	https://professional.heart.org/en/research-programs/aha-fundingopportunities/career-developmentaward Early-career phase
The American Association for Thoracic Surgery Surgical Investigator Program	Up to $50,000/y for up to 2 y, 2-y proposals are preferred	Can be in the process of obtaining certification by the appropriate Thoracic Surgery Board in the United States, Canada, or an international equivalent. Within their first 7 y of practice. The focus should be on aortic and valvular heart disease. All candidates who submit eligible applications will be considered for all available AATS Foundation Surgical Investigator funding	
AATS Foundation Surgical Investigator Award		Open to proposals from both cardiac and thoracic specialties	All career phases
Lawrence H. Cohn Surgical Investigator Award		Open to cardiac proposals	All career phases
Timothy J. Gardner Surgical Investigator Award		Open to cardiac proposals	All career phases
Jack A. Roth Surgical Investigator Award		Open to thoracic proposals focused on surgical oncology	All career phases
Tirone E. David Surgical Investigator Award		Open to cardiac proposals focused on aortic and valvular heart disease	All career phases
National Institutes of Health K Award	Up to $100,000/y	Postdoctoral or early faculty level, working toward research independence, and the need for mentored research and career development	https://researchtraining.nih.gov/programs/career-development/K01 Early-career phase
National Institutes of Health R Award		Investigators who can demonstrate the ability to conduct independent research	https://grants.nih.gov/funding/activitycodes/R01 All career phases

∗ Surgery by the start of the grant and within 7 years of their first faculty appointment.
